# Induction of Polyacetylene to a Chiral Smectic Liquid Crystal–Chiral Direct Conversion

**DOI:** 10.3390/polym12071547

**Published:** 2020-07-13

**Authors:** Akiko Yatsu, Takuya Yonehara, Hiromasa Goto

**Affiliations:** Department of Materials Science, Faculty of Pure and Applied Sciences, University of Tsukuba, Tsukuba Ibaraki 305-8573, Japan; ruri9314@gmail.com (A.Y.); s2030069@s.tsukuba.ac.jp (T.Y.)

**Keywords:** chiral dopant, chiral smectic, direct conversion, electro-dopant, ESR, liquid crystal, mono-substituted polyacetylene

## Abstract

The synthesis of polyacetylene-bearing pyrimidine-type three-ringed mesogenic core exhibiting smectic C (SmC) characteristics was conducted. Gas-phase iodine doping of the polymer provided evidence of chemical interaction between the polyene and iodine, which acted as an electron acceptor. The side-chain fluorine atom tilted the mesogen moiety to form SmC as a tilted liquid crystal. The addition of a small amount of chiral inducer yielded SmC* of the polymer as the chiral version of SmC. The liquid crystallinity and electronic properties of the π-conjugated chiral liquid crystal polymer with a helical structure were evaluated.

## 1. Introduction

In 1986, a directly aligned polyacetylene film using liquid crystal (LC) as the polymerization solvent was synthesized [1]. The polymer, which was prepared via linear orientation, exhibited high electrical conductivity when subjected to iodine doping. The side-chain-type LC π-conjugated polymer was studied to better understand the molecular alignment of π-conjugated polymers through liquid crystallinity. The side-chain array in the LC state induced spontaneous orientation of the polymer [2,3], thereby improving the coplanarity of the main chain. However, the large size of the LC group (mesogen) resulted in the torsion of the main chain, which deformed the coplanarity of the π-conjugation. From the viewpoint of liquid crystal science, the π-conjugated polymer with liquid crystallinity is quite an intriguing issue because collaboration between the rigid main chain of the π-conjugated system and the side-chain arrangement resulted in an unprecedented LC performance [4,5,6,7,8,9,10]. Additionally, the choice of functional LC molecules for subsequent introduction to conjugated polymers, including LCs that displayed high dielectricity, chirality, and photo-induced isomerization, resulted in LCs with unique physical functions. Aromatic type liquid crystalline π-conjugated polymers showed emission function, electrical conductivity, and good electrochemical properties, making them ideal for energy applications as batteries [11,12,13,14].

In this research, we develop a new, functional, LC-conjugated polymer that incorporated principles used in the development of conducting polymers, LC-conducting polymers, chiral-conjugated polymers, and polymerization in LC. The use of a chiral inducer for generating LC polyacetylene derivatives with nonchiral smectic C (SmC) LC blends is conducted in this study to obtain induced type chiral smectic liquid crystals that exhibited SmC* characteristics.

Herein, the synthesis of the monosubstituted LC polyacetylene derivative is conducted by introducing a tricyclic liquid crystal substituent that possesses a fluorine atom on the side chain. Steglich esterification is performed to prepare the monomer. Polymerization is carried out with a rhodium complex catalyst to obtain the corresponding brown polymer that exhibits schlieren and a fingerprint texture of smectic C (SmC) when observed via polarizing optical microscopy.

This paper reports the successful synthesis of the SmC–liquid crystal–polyacetylene (SmC–LC–poly(Ac–Pyr)), and induction of the formation of SmC* with a helical structure by the addition of a small amount of a chiral molecule.

## 2. Results

### 2.1. Synthesis of the Monosubstituted Polyacetylene

**Scheme 1:**
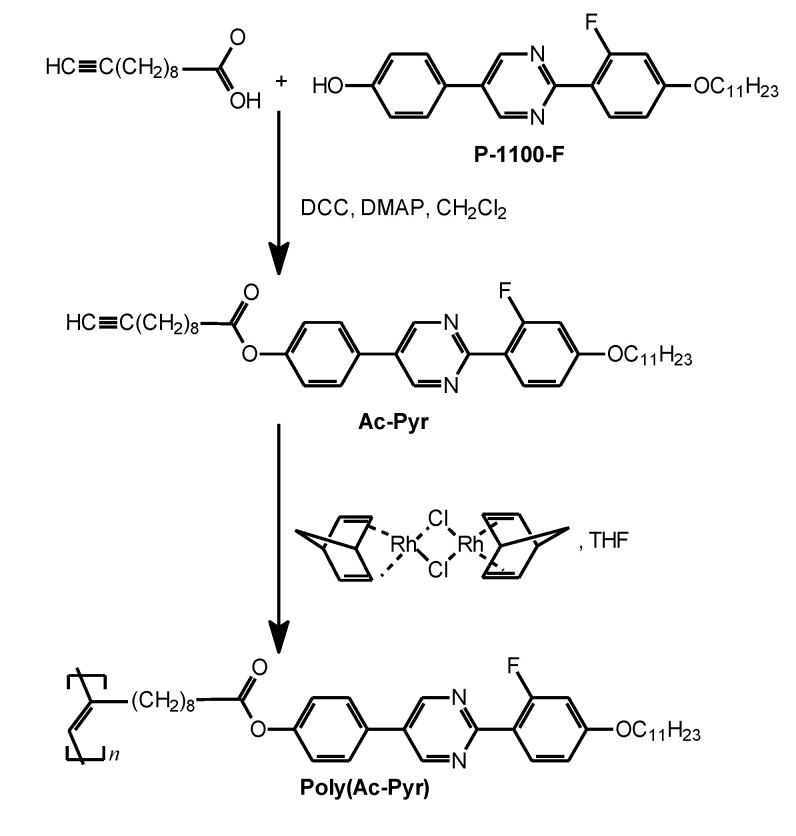
Synthesis of the polyacetylene–bearing fluorinated three-ringed pyrimidine type mesogen. DCC: dicyclohexylcarbodiimide. DMAP: 4-dimethyaminopyridine. THF: tetrahydrofuran.

Scheme 1 shows the synthetic route for the polyacetylene bearing fluorinated three-ringed pyrimidine type mesogen. The mesogen core with the hydroxy group, which was abbreviated as P-1100-F (i.e., P: pyrimidine, 11: carbon number of the terminal alkyl group, 0: oxygen, F: fluorine), was provided by Midori Kagaku (Midori Chemical Industry, Tokyo, Japan). First, P-1100-F was subjected to Steglich esterification with 10-undecynoic acid using dicyclohexylcarbodiimide (DCC) and 4-dimethyaminopyridine (DMAP) in CH_2_Cl_2_. A mixture of P-1100-F (0.49 g, 1.14 mmol), 10-undecynoic acid (0.208 g), DCC (0.24 g), and DMAP (0.14 g) in CH_2_Cl_2_ was stirred at rt. After 24 h, the reaction mixture was filtered, and the solvent was removed under vacuum to give the product that, after recrystallization from acetone, yielded white crystals (0.56 g) with an *Rf* of 0.71 (TLC, CH_2_Cl_2_) in 81.7% yield. This monomer was abbreviated as Ac–Pyr. Here, Ac and Pyr denoted acetylene and the pyrimidine derivative mesogen, respectively. Next, Ac–Pyr (57.5 mg) and [Rh(NBD)Cl]_2_ (NBD = norbornadiene, 2.33 mg) in *N*,*N*-dimethylformamide (DMF, 1 mL) were stirred for 24 h. The mixture was poured into a large volume of methanol. The precipitate was collected via filtration and dried under reduced pressure to yield the polymer product as a brown powder (28 mg) in 48.7% yield and was abbreviated as poly(Ac–Pyr). Number average molecular weight (*M*_n_) and weight average molecular weight (*M*_w_) vs. polystyrene standard are 9680 g/mol and 11,260 g/mol (eluent: THF), respectively. Dispersity of the polymer is ca. 1.2.

### 2.2. Characterization of Poly(Ac-Pyr)

#### 2.2.1. Infrared Absorption

**Figure 1:**
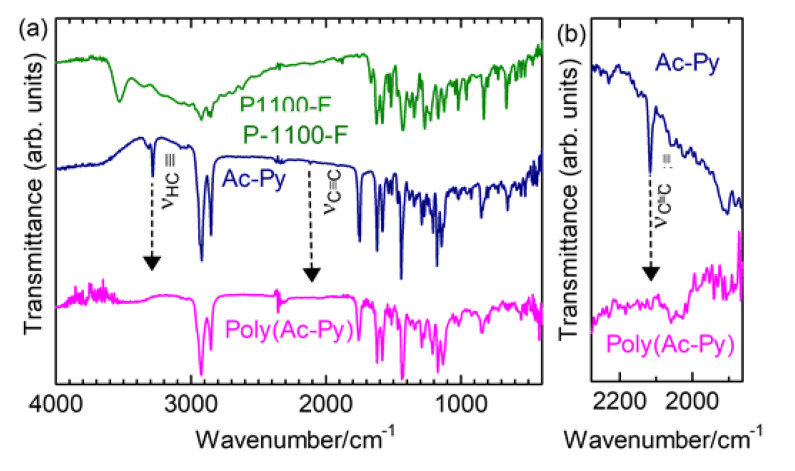
(**a**) Fourier-transform infrared (FT–IR) absorption spectra of P-1100-F (mesogen), Ac–Pyr (monomer, monomer, Ac-Py in the Figure), and poly(Ac–Pyr) (polymer, poly(Ac-Py) in the Figure). (**b**) Magnification of the IR absorption bands in the region of n_C≡C_.

Figure 1 shows the Fourier-transform infrared absorption (FT-IR) spectra of P-1100-F (mesogen core), Ac–Pyr (monomer), and poly(Ac–Pyr) (polymer). As noted, Ac–Pyr exhibited no absorption band due to the OH group present. The IR spectrum of the polymer had no absorption characteristics related to the acetylenic moiety in the monomer (≡C–H stretching at 3280 cm^−1^, C≡C stretching at 2117 cm^−1^, Figure 1b), and the IR spectrum of the polymer was similar to that of the corresponding monomer. This result indicated that the synthesis of the polymer had been successful.

#### 2.2.2. NMR

**Figure 2:**
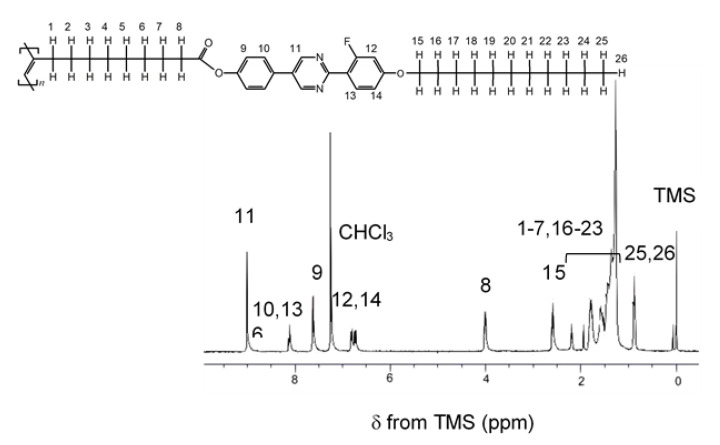
^1^H NMR spectrum of poly(Ac–Pyr) in CDCl_3_.

Figure 2 shows the ^1^H nuclear magnetic resonance (^1^H NMR) spectrum of poly(Ac–Pyr). Here, the aromatic protons in the vicinity of the fluorine atom were observed in the low magnetic range, whereas the protons of the alkyl group were located in the high magnetic range. The olefinic proton of the *cis* form of poly(Ac–Pyr) was not observed, indicating that poly(Ac–Pyr) was a *trans*-rich polyacetylene.

### 2.3. Physical Properties

#### 2.3.1. Optical Texture

**Figure 3:**
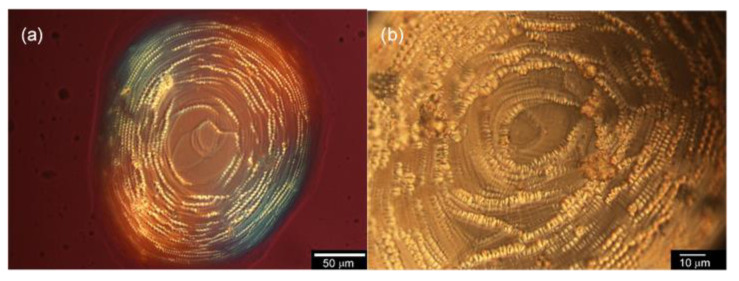
Polarizing optical microscopy (POM) images of P-1100-F at 99 °C. (**a**) Insertion of a gypsum first-order red plate. (**b**) POM image of P-1100-F at higher magnification.

Figure 3 shows the optical textures of P-1100-F as a mesogen group at 99 °C when observed via polarizing optical microscopy (POM). The POM image of P-1100-F with the insertion of a gypsum first-order red plate can be seen in Figure 3a. The fan-shaped smectic A (SmA) domain aggregate was circular with wire-like formations. The magnified image shows these spiral wires consisting of the sequential wedge form of SmA (Figure 3b).

**Figure 4:**
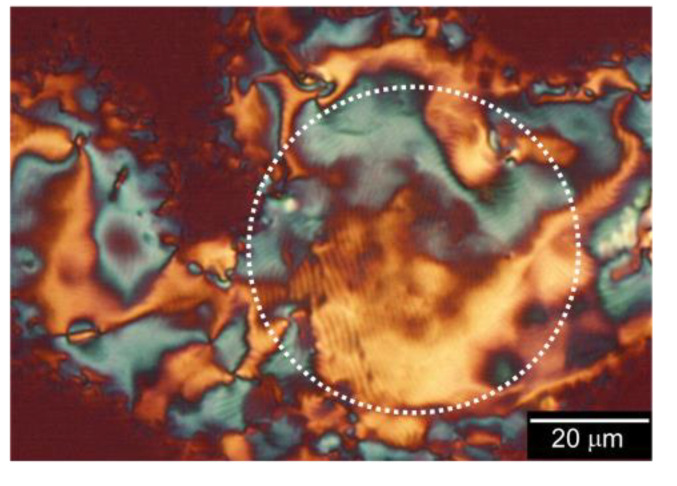
POM image of poly(Ac–Pyr) with the insertion of a gypsum first-order red plate under cross-Nicol conditions. Inside the dotted circle shows the fingerprint–textured stripe.

Figure 4 shows the results of optical microscopy of poly(Ac–Pyr) with the insertion of a gypsum first-order red plate under cross-Nicol conditions at 63 °C in cooling. The LC structure was maintained at r.t. as a solid. The POM image of poly(Ac–Pyr) exhibited a four-blushed schlieren texture and a partial fingerprint structure (shown in the dotted circle in the picture) due to the formation of SmC.

#### 2.3.2. Modification of the Poly(Ac-Pyr) SmC Phase

**Figure 5:**
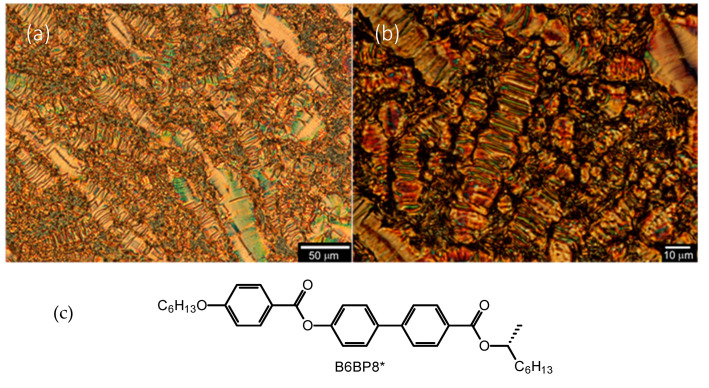
POM images of the SmC*–LC–poly(Ar–Pyr) blend with the chiral inducer, C6BPC8*, at different sections of the same sample. (**a**) Low magnification. (**b**) High magnification image. (**c**) chemical structure of B6BP8* as a chiral inducer.

The addition of a chiral inducer (chiral dopant), which was abbreviated as B6BP8*, to the achiral SmC produced the chiral smectic C (SmC*), Figure 5c.

**Figure 6:**
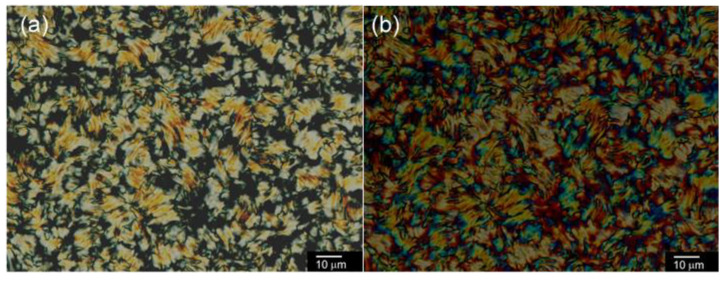
(**a**) POM images of the SmC*–LC–poly(Ar–Pyr) blend with the chiral inducer, C6BPC8*. (**b**) Circular polarized differential interference contrast optical microscopy (C-DIM) image of the SmC*–LC–poly(Ar–Pyr) blend with C6BPC8*, the same region as (**a**).

**Figure 7:**
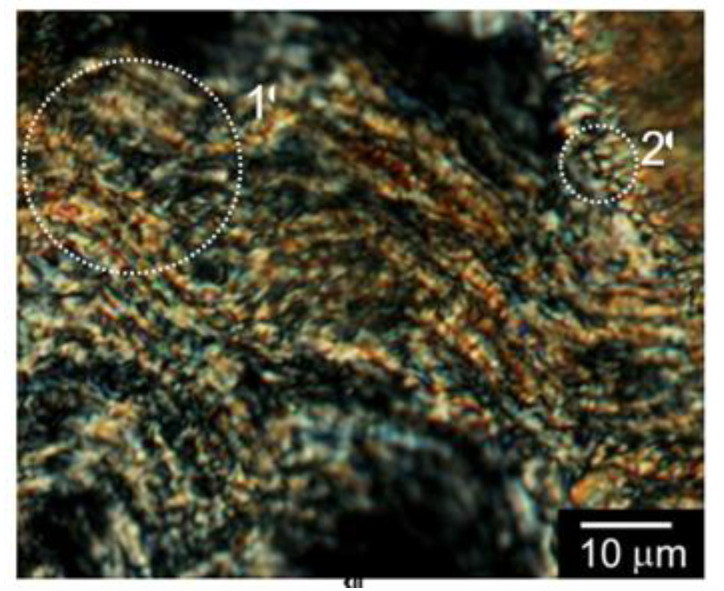
POM image of cast film of the SmC*–LC–poly(Ar–Pyr) blend with the chiral inducer prepared from THF solution. 1: Fingerprint-structure. 2: Diamond structure.

The chiral molecule of the three-ring system with an optically active, terminal alkyl group (0.25 mg) was mixed with poly(Ac–Pyr) (3.03 mg) to obtain the SmC*–LC–poly(Ac–Pyr) blend. Figure 5a,b shows the POM images of the SmC*–LC–poly(Ac–Pyr) blend containing a striped fan-shaped texture that was typical for SmC*. The distance between the stripes corresponds to the helical pitch. Figure 6a displays a different sample region of the blend, showing a broken fan-shaped texture. Figure 6b depicts a circular polarized differential interference contrast optical microscopy (C-DIM) image of the SmC*–LC–poly(Ar–Pyr) blend with C6BPC8*, the same region as (a). This image enhances stripes with color. Further, cast film of the polymer blend from THF partly shows birefringence under the POM, as shown in Figure 7. In the smectic oily streaks, region 1 in Figure 7 partly exhibits a fingerprint texture, and region 2, a diamond structure of smectic LC consisting of four-domains, which are generally observable for the smectic phase. This observation demonstrates that the polymer blend exhibits lyotropic LC (LC in solvents). After the evaporation of THF from the solution, the polymer blend was solidified while maintaining the smectic LC structure. Therefore, the SmC*–LC–poly(Ar–Pyr) blend with C6BPC8* has amphotropic liquid crystallinity (both thermotropic and lyotropic LC).

**Figure 8:**
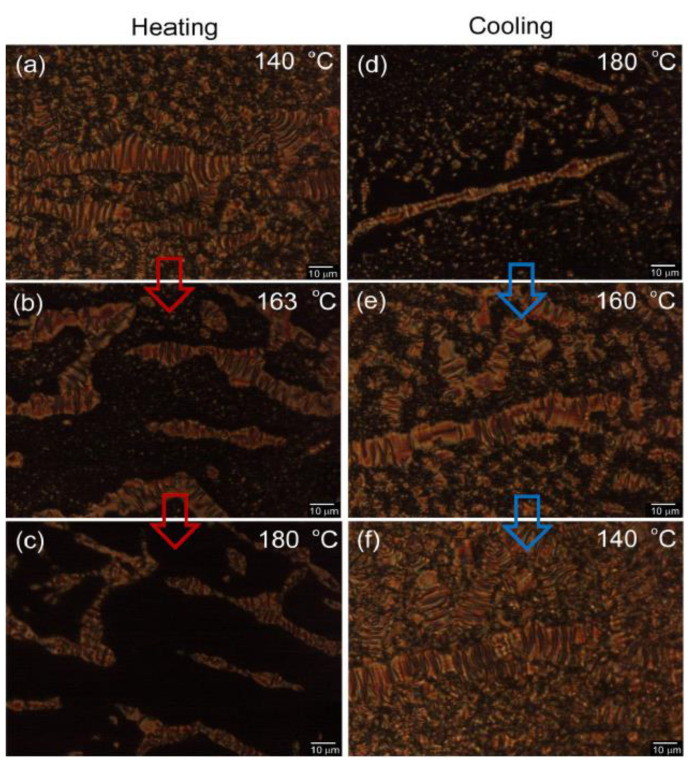
POM images of the SmC*–LC–poly(Ar–Pyr) blend with the chiral inducer on heating (**a**–**c**) and cooling (**d**–**f**).

**Figure 9:**
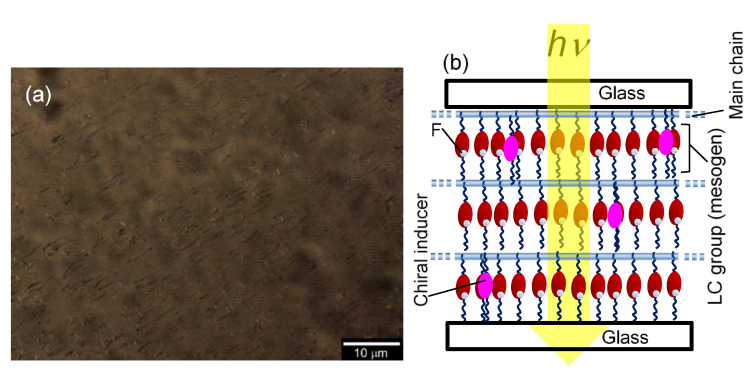
The POM image of (**a**) the pseudo-homeotropic-oriented (i.e., the vertical orientation against the glass cell surface) section of the SmC*–LC–poly(Ac–Pyr) blend with the chiral inducer. (**b**) Homeotropic alignment.

**Figure 10:**
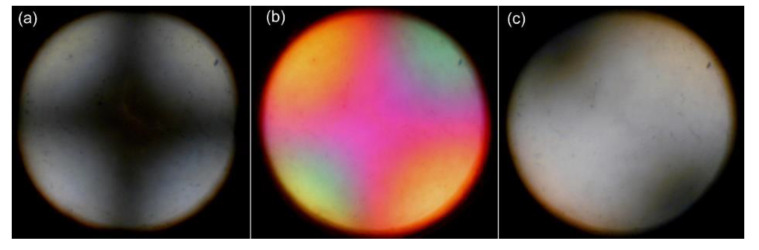
Conoscopic observations for the perpendicularly arranged section of the SmC*–LC–poly(Ac–Pyr) blend with the chiral inducer in the glass cell as the pseudo-homeotropic alignment. (**a**) Cross-Nicol condition. (**b**) A gypsum first-order red plate, which was inserted under cross-Nicol conditions, showing Maltase cross color changes. (**c**) The insertion of a ¼-wavelength plate.

The analysis showed that the heating and cooling process changed the optical texture, as seen in Figure 8. Here, the striped fan-shaped texture melted once heated, and the melted domains underwent a phase transition to the isotropic phase at 180 °C (Figure 8a–c). The striped fan-shaped domains of SmC* grew when cooled (Figure 8d–f). The striped domain structure remained fixed at rt. The SmC*–LC molecule is known as an orientation polarization type ferroelectric LC. Since the high viscosity of the polymer is known to slow the molecule’s response to external electric fields, it is difficult to conduct dielectric measurements using the Sawyer–Tower method. The hysteresis curve, which was due to the ferroelectricity of the polymer, may have obtained if certain conditions were first achieved, namely, surface stabilization of the polymer in the measurement cell, the proper external orientation, and the use of a low frequency for measuring the sample. The orientation of the LC blend of the poly(Ac–Pyr) sample with the chiral inducer was achieved via shear stress using the application of a high voltage or a magnetic field to the solid film, thereby facilitating ferroelectricity through the generation of SmA-like alignment from SmC*. The alignment of the polymer’s dipole moment was conducted to achieve ferroelectricity. Therefore, the fingerprint texture changed to a non-characteristic optical texture on completion of alignment and the occurrence of surface stabilization. As a tentative step, pseudo-homeotropic alignment of the sample was attempted and observed in a glass cell, as shown in Figure 9a. Note that the tilt directors of the SmC* molecules in the homeotropic alignment averaged at zero (Figure 9b). The texture was viewed with an average perpendicular alignment of the SmC* molecules. The cross-Nicol lines observed using the conoscope exhibited Maltese cross characteristics (Figure 10a). The blue first and third quadrants, as well as the yellow second and fourth quadrants after the insertion of the gypsum first-order red plate, demonstrated that the polymer is an optically positive uniaxial material (Figure 10b). The insertion of a 1/4-wavelength plate produced black second and fourth quadrants (Figure 10c). The symmetry of the Maltese cross indicated that the mesogenic side chains had no apparent tilt-angle concerning the glass substrate, indicating that the polymer had been aligned perpendicular to the SmA*-type structure under the guidance of the director, thereby exhibiting ferroelectricity. However, a dynamic control of this physical trait was not conducted due to the high viscosity of the polymer, although the blended LC generally displayed low viscosity. Thus, this polymer can be applied for optical memory devices because of the retention of the orientation state with good contrast.

#### 2.3.3. Dynamic Scanning Calorimetry

**Figure 11:**
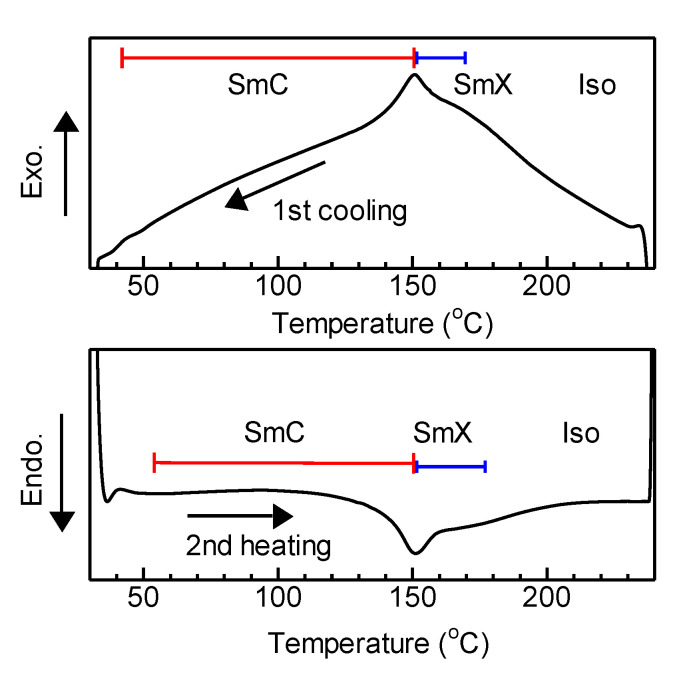
Dynamic scanning calorimetry conducted for poly(Ac–Pyr) in the first cooling and second heating processes.

Dynamic scanning calorimetry (DSC) was conducted on the pure poly(Ac–Pyr) sample, as shown in Figure 11, after the first heating process. Both the first cooling and the second heating processes exhibited DSC signals that corresponded to the mesophase of SmC. The phase transition of poly(Ac–Pyr) followed the order, glassy 51(53) SmC 151(150) SmX 171(166) Iso in °C, where the numbers in parentheses represent the phase transition temperatures of the cooling process. The inflection points observed in the DSC spectrum at 177 °C (heating) and 166 °C (cooling) indicated that a phase transition from the isotropic to unknown LC phase, which may be SmA, had occurred and was denoted as SmX.

### 2.4. Modification of the Poly(Ac–Pyr)

#### 2.4.1. Influence on Polyacetylene Bandgap by Iodine Doping

**Figure 12:**
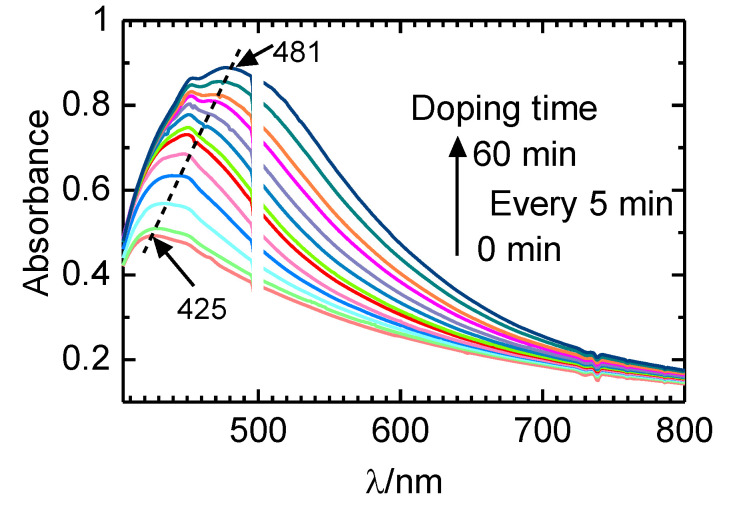
Change in the optical absorption of poly(Ac–Pyr) after gas-phase iodine doping at 5-min intervals.

In situ gas-phase doping of the iodine vapor for the polymer was conducted to examine the effect exerted by acceptor doping processes using polyene as the main chain to evaluate the electronic function (Figure 12). As the gas-phase iodine-doping process proceeded, the absorption band observed at 425 nm is attributed to the electronic transition of the main chain as it gradually shifted to the long-wavelength region, thereby confirming the feasibility of the chemical doping process of the polymer. The band-edge bandgap of poly(Ac–Pyr) was 1.8 eV, which is in the range of the semiconductor. Iodine doping of the polymer changed the band-edge, implying the appearance of a mid-gap. However, this polymer had no distinct new band due to the generation of charged species. Therefore, this red-shift of the optical absorption of poly(Ac–Pyr) with iodine doping implies the formation of the iodine–polyene chemical complex containing a small amount of charge species.

**Figure 13:**
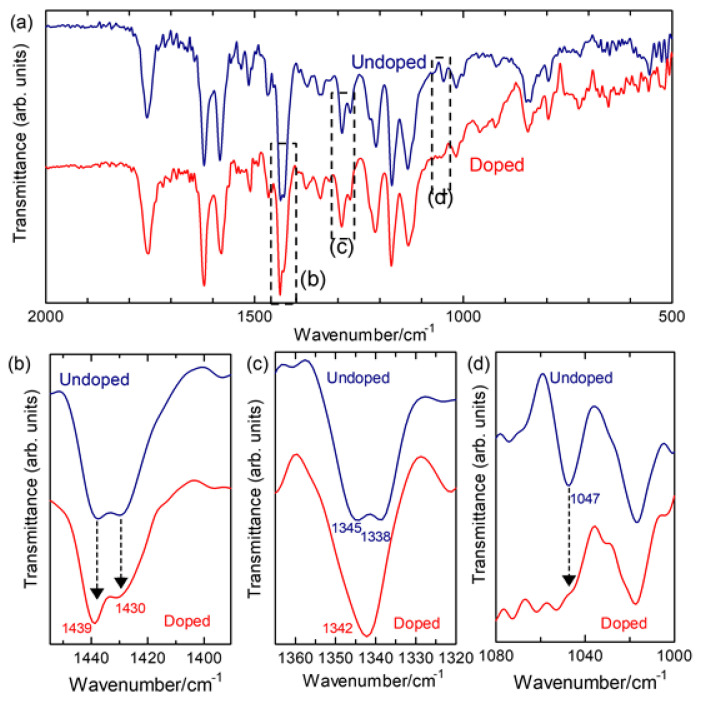
IR absorption spectra of poly(Ac–Pyr). (**a**) IR absorption of poly(Ac–Pyr) before and after iodine doping. (**b**–**d**) Magnification of the IR absorption spectra of poly(Ac–Pyr) before and after iodine doping.

IR absorption spectroscopy results of poly(Ac–Pyr) before and after iodine doping are shown in Figure 13a. The absorption bands observed at 1439 cm^−1^ increased after iodine doping had been undertaken (Figure 13b). The absorption band observed at 1342 cm^−1^ was attributed to the delocalized C=C bond as solitons are generally observable at 1380 cm^−1^ for non-substituted polyacetylene [15]. The disappearance of the absorption signal at 1047 cm^−1^ was observed for doped poly(Ac–Pyr), indicating the gas-phase iodine, as an electron acceptor, changed the structure of poly(Ac–Pyr) by altering the electronic state.

**Figure 14:**
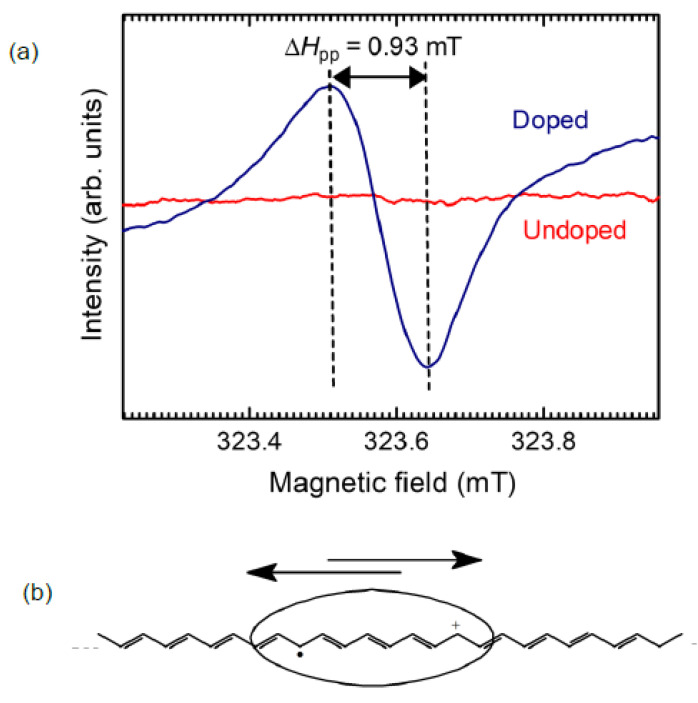
Electron spin resonance (ESR) absorption spectra of poly(Ac–Pyr (**a**). Solitons in polyacetylene (**b**).

Gas-phase doping of iodine for poly(Ac–Pyr) exhibited electron spin resonance absorption (Figure 14a). The spin species was derived from radical cations on the main chain that had been generated by the iodine vapor as the electron acceptor. The as-prepared undoped sample displayed no signals in the ESR because there were no charge carriers. The charge carriers of the radical cations in the doped poly(Ac–Pyr) are referred to as polarons (Figure 14b). The physical behavior of the charge carriers of the polyacetylene was similar to that of solitons [16,17,18]. The peak-to-peak line width (Δ*H*_pp_) was narrow (0.93 mT), indicating that the charge carriers were delocalized along the main chain. The iodine doping of poly(Ac–Pyr) confirmed the doping effect on the polymer. The sensitive ESR measurement demonstrated the generation of a small portion of charge species in the main chain upon iodine doping, although no clear new doping band appeared in the UV-Vis.

#### 2.4.2. Inducting Uniaxial Alignment of Chiral Smectic LC

**Figure 15:**
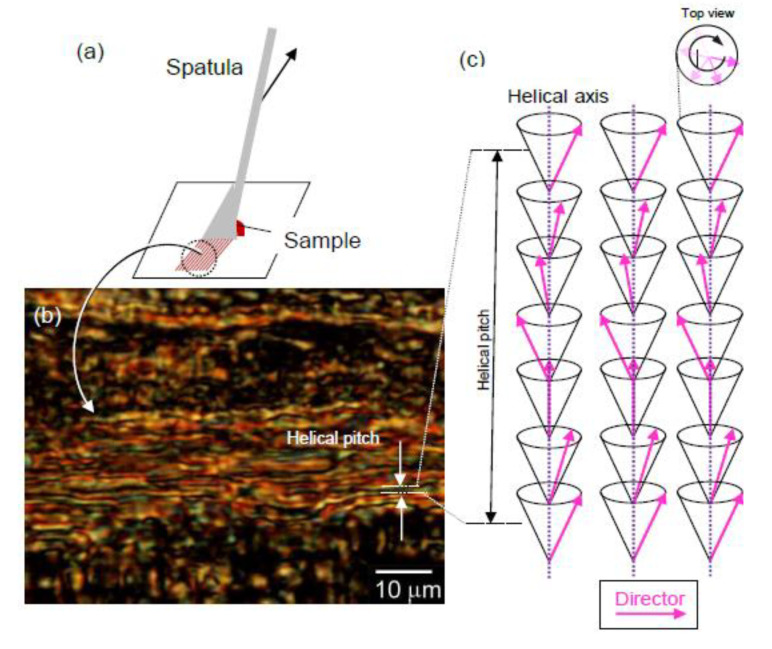
(**a**) Mechanical orientation of the SmC*–LC–poly(Ac–Pyr) blend using a spatula. (**b**) POM image of the oriented SmC*–LC–poly(Ac–Pyr) blend. (**c**) The helical superstructure of the SmC* phase.

The mechanical orientation of the SmC*–LC–poly(Ac–Pyr) blend was conducted with a spatula, as shown in Figure 15a. The shear stress produced thin, oriented samples of poly(Ac–Pyr) that exhibited uniaxial alignment. The SmC*–LC domains contained striped lines that were observable via POM analysis at r.t. (Figure 15b). In this case, the polymer film, which was solid and maintained the LC order at rt, was referred to as the oriented chiral SmC*, with a helical feature that was arranged counter to the perpendicular direction of the stripes. When a high shear rate was applied to the sample, the destruction of this helical feature resulted in the formation of SmA* with no helical arrangement having uniaxially oriented polarization for exhibiting ferroelectricity. Figure 15c shows the helical arrangement of the poly(Ac–Pyr) molecules represented with the accompanying LC directors. In the top view, the LC director has rolled across the SmC* layer to establish interlayer helicity. The distance between the two stripes observed in Figure 5b corresponds to one pitch of the helical structure of poly(Ac–Pyr) that was influenced by the chiral inducer. The pitch was, thus, ca. 1.1 µm at r.t.

## 3. A Plausible Structure for the Chiral Phase

**Figure 16:**
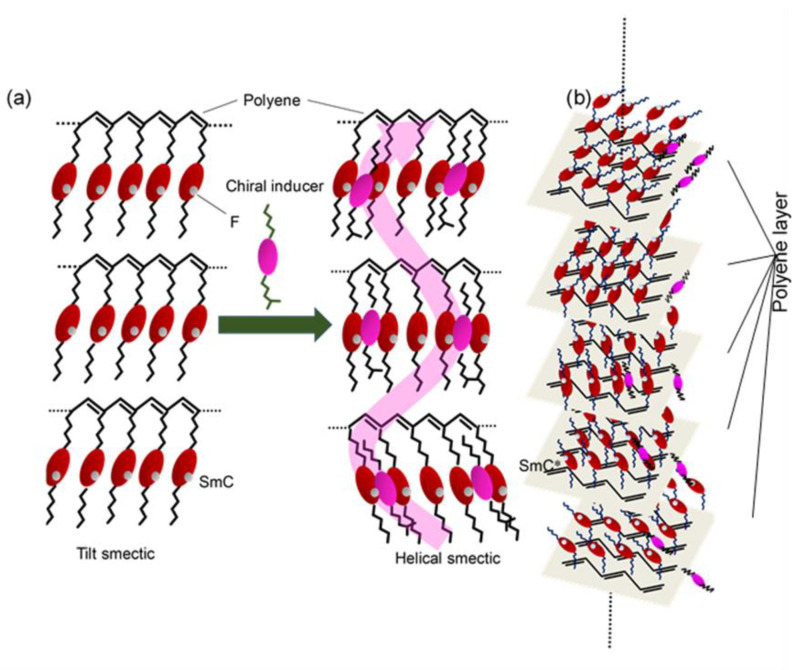
(**a**) Induction of SmC* from SmC via the addition of the chiral inducer. (**b**) The helical superstructure of SmC*–LC-poly(Ac–Pyr) with a polyene layer.

The addition of the chiral inducer resulted in the formation of the helical arrangement of poly(Ac–Py) from an achiral, simple, tilted SmC (Figure 16a). The SmC*–LC blend formed a layered structure, whereas the directors of the mesogen rotated to form helices. Since the flexibility of the π-conjugated polymers is generally lower than that of polyolefins, using the polyene as the main chain led to the formation of a layered structure with the aid of the LC arrangement of the SmC* phase (Figure 16b). The direct addition of a small number of chiral inducers (chiral dopants) to achiral smectic LCs for the formation of a steady helical structure can be referred to as "Direct Conversion" to chiral polymeric LC. This polymer blend shows counterclockwise (left-handed) optical rotation based on the helical structure (Figure S1, Supplementary Materials).

The striped structures of poly(Ac–Py) with no chiral inducer may have facilitated the helical arrangement of SmC, as evidenced by the observable fingerprint texture, shown in Figure 4. Generally, achiral SmC forms simple, tilted arrangements with layered structures. By chance, we discovered that the achiral SmC–LCs occasionally exhibited a fingerprint texture when observed via the POM. Here, mixed helicity with a clockwise and a counterclockwise helical arrangement of the achiral SmC–LCs was preferentially generated rather than the simple molecular tilted form of SmC. The combination of right-handed and left-handed helical arrangements (tentatively referred to as racemic helicity) of the achiral SmC generated helical structures that could partly exhibit the above mentioned fingerprint structures like those noted in SmC*.

## 4. Conclusions

We synthesized a polyacetylene derivative bearing a pyrimidine-type three-ringed mesogenic core that exhibited SmC characteristics. Gas-phase iodine doping of the polymer induced chemical interaction between the polyene and iodine, which served as the electron acceptor, to form charge carrier solitons. The fluorine atom on the side chain of the mesogen altered the mesogen itself, thus, resulting in a tilted LC. The addition of a small number of chiral inducers (dopants) yielded SmC*, which exhibited a helical structure similar to that of the chiral version of SmC, as Direct Conversion to chiral polymeric LC.

## 5. Instruments

Molecular weights of the polymers were determined by GPC against polystyrene standards using THF as an eluent with a 5 μm-MIXED-D column (Polymer Laboratories, Church Stretton), a PU-980 HPLC pump (JASCO, Tokyo, Japan), and an MD-915 multiwavelength detector (JASCO, Tokyo, Japan). ^1^H NMR spectrum was recorded on a JNM-ECS-400 NMR Spectrometer (JEOL, Tokyo). All signals were reported in ppm with the internal TMS (tetramethylsilane) at 0.00 ppm. FT-IR absorption spectra were obtained with an FT/IR-4600 (JASCO) using the KBr method. All spectra were recorded between 400 and 4000 cm^−1^ with a resolution of 4 cm^−1^. POM observation was conducted with an Eclipse LV100 high resolution polarizing microscope (Nikon, Tokyo, Japan). UV-Vis absorption spectra were recorded on a V-630 UV-Vis spectrophotometer (JASCO, Tokyo, Japan). DSC measurements were performed with an EXSTAR7000 (Seiko Instruments Inc., Chiba, Japan). ESR measurements were conducted with a JES-TE-200 (JEOL, Tokyo, Japan) with X-band.

## Supplementary Materials

The following are available online at https://www.mdpi.com/2073-4360/12/7/1547/s1, Figure S1: Optical rotatory dispersion spectrum of SmC*–LC–poly(Ac–Pyr) blend. Inset shows linear dichroism of the blend film (red line).
