# Supplementary material for: Induction of Polyacetylene to a Chiral Smectic Liquid Crystal–Chiral Direct Conversion
Source: Polymers (Basel). 2020 Jul 13;12(7):1547. doi: 10.3390/polym12071547 (PMC7407238; doi:10.3390/polym12071547)
Supplement: Supplementary file 1 [file polymers-12-01547-s001.pdf]

polymers

MDPI

Brief Report

Induction of Polyacetylene to a Chiral Smectic Liquid Crystal Chiral Direct Conversion

Akiko Yatsu, Takuya Yonehara and Hiromasa Goto*

Department of Materials Science, Faculty of Pure and Applied Sciences, University of Tsukuba, Tsukuba, Japan.
*Corresponding author, e-mail; gotoh@ims.tsukuba.ac.jp
Received: 14 June 2020; Accepted: 07 July 2020; Published: date

Figure S1 displays optical rotarory dispesion (ORD) spectroscopy measurement result, indicating the polymer blend film with SmC* order shows left direction optical rotation. Linear dichroims element of the blend sample for the ORD mesurement is negligible (inset of Figure S1) because random orientation of the polymer. A chiral inducer with anitipode chirality may induce opposite helical direction for the substituents of the polymer to show right direction optical rotation.

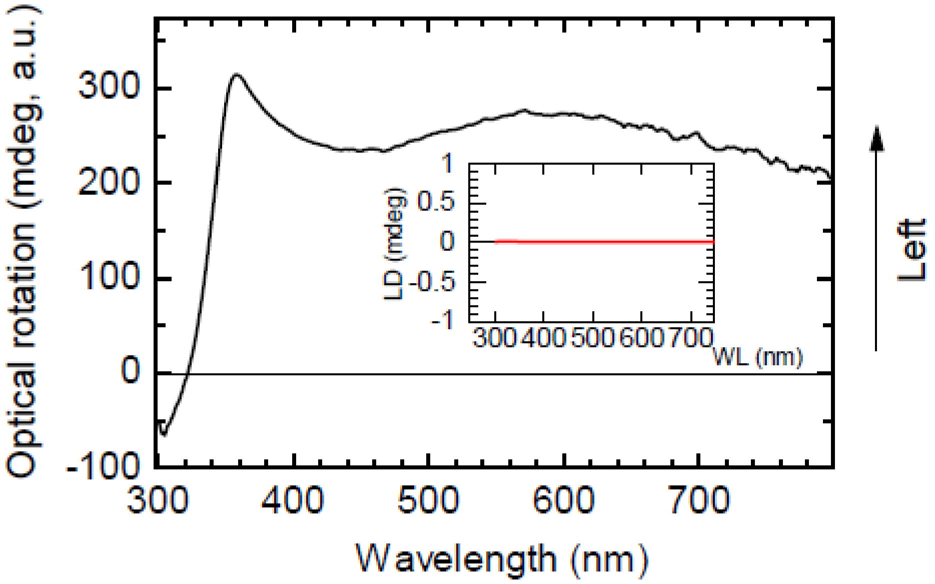

Figure 1. Optical rotatory dispersion spectrum of SmC*–LC–poly(Ac–Pyr) blend. Inset shows linear dichroism of the blend film (red line).

© 2018 by the authors. Submitted for possible open access publication under the terms and conditions of the Creative Commons Attribution (CC BY) license (http://creativecommons.org/licenses/by/4.0/).
